# Spindle cell carcinoma: the general demographics, basic clinico-pathologic characteristics, treatment, outcome and prognostic factors

**DOI:** 10.18632/oncotarget.18017

**Published:** 2017-05-19

**Authors:** Lei Feng, Deng Cai, Alanuer Muhetaer, Yin-Long Yang, Fei Ren, Mumingjiang Yishake, Hao Zhang, Yuan Fang, Alimujiang Wushou

**Affiliations:** ^1^ Department of Oral and Maxillofacial Surgery, Shanghai Stomatological Hospital, Fudan University, Shanghai 200001, China; ^2^ Department of Thoracic Surgery, Fudan University Shanghai Cancer Center, Shanghai 200032, China; ^3^ Department of Gynaecology, Zhongshan Hospital, Fudan University, Shanghai 200000, China; ^4^ Department of Breast Surgery, Fudan University Shanghai Cancer Center, Shanghai 200032, China; ^5^ Department of Pathology, Fudan University Shanghai Cancer Center, Shanghai 200032, China; ^6^ Department of Orthopedics Surgery, Zhongshan Hospital, Fudan University, Shanghai 200000, China; ^7^ Department of Epidemiology and Biostatistics, Shanghai Stomatological Hospital, Fudan University, Shanghai 200001, China; ^8^ Department of General Surgery, Zhongshan Hospital, Fudan University, Shanghai 200000, China

**Keywords:** spindle cell carcinoma, SEER analysis, prognostic factors

## Abstract

**Background:**

Owing to the rarity, the general demographics, basic clinico-pathologic features, management, outcome and prognostic factors of spindle cell carcinoma (SpCC) were unexplored.

**Methods:**

A SEER analysis was performed with 2336 cases (1973-2016).

**Results:**

A peak incidence occurred at 70~80 years without any gender predominance and 83.13% occurred in white people. The respiratory system was mostly affected tumor site (35%). Significant overall survival (OS) and disease specific survival (DSS) were found differentiated in gender, age, marital status, primary tumor location, AJCC stage, T stage, N stage, M stage, pathologic grade and treatment modality. In the multivariate Cox model, the age > 69 years (Hazard ratio [HR] = 1.427 for OS, *P* = 0.01 and HR = 1.491 for DSS, *P* = 0.003; Reference [Ref] age ≤ 69 years), tumor location in respiratory system (HR = 1.550 for OS, *P* = 0.041 and HR = 1.561 for DSS, *P* = 0.04; Ref: digestive system), N2 stage (HR = 1.962 for OS, *P* = 0.006 and HR = 1.982 for DSS, *P* = 0.004; Ref: N0 stage) and AJCC stage IV (HR = 4.601 for OS, *P* = 0.000 and HR = 5.107 for DSS, *P* = 0.000; Ref: stage I) were independently associated with worse OS and DSS.

**Conclusions:**

SpCC mostly occurred in white people at 70~80 years old without predominance in any gender. The respiratory system was mostly affected site. The patient's age, primary tumor location, AJCC stage were independent prognostic indicators for both DSS and OS of SpCC.

## INTRODUCTION

Spindle cell carcinoma (SpCC), also called sarcomatoid carcinoma, is a relatively uncommon and it has histologic, cytologic and molecular properties of both epithelial and mesenchymal tumors [1, 2]. Thus, it presents heterogeneous pathologic features, clinical behavior and prognosis [3–5]. In the past, it was easily be misdiagnosed because of the unfamiliarity. Nowadays, sophisticated pathologic molecular techniques has made the diagnosis of SpCC reliable [6].

For SpCC, there is no standard treatment protocol available from previously published studies. Treatment modalities of SpCC has varied across previous reports [7, 8]. Although the mainstay treatment for SpCC is surgery, it varied from local excision to radical surgery [5, 7, 9]. The addition of radiotherapy or chemotherapy to treatment protocol has also varied. In most cases, radiotherapy and chemotherapy were added after surgery considering various common cancer adverse prognostic factors such as unconfirmed surgical margins, poor pathologic differentiation and advanced tumor stage etc [8, 10–12].

Regarding general demographics, basic clinico-pathologic features, management strategies and prognosis, there is lack of instructive data to guide SpCC management and to evaluate prognosis, owing to the rarity of the disease. We assume that a nationwide population-based cohort may provide an opportunity to evaluate trends in overall demographic features, basic clinico-pathologic characteristics and compare treatment modalities and outcome of SpCC. Thus, we performed a generalized investigation of all SpCC cases registered in the Surveillance, Epidemiology, and End Results (SEER) from 1973 to 2016.

## RESULTS

**Figure 1:**
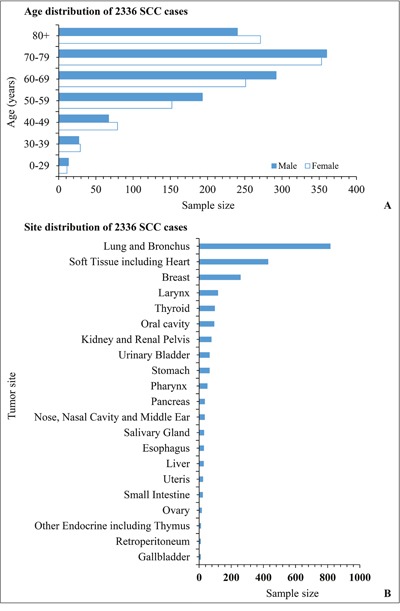
The distribution of age **(A)** and primary tumor location **(B)** of all SpCC cases registered in the SEER database.

A total of 2336 cases with diagnosis of SpCC were found in the SEER database from 1973 to 2016. The median follow-up time was 32.5 months (range, 1-437 months). Gender distribution was nearly equal including 1191 men and 1145 women. Majority of SpCC occurred in white people (83.13%, 1942/2336). A peak incidence occurred during the seventh decade of life. SpCC could be found in almost any site of the body. However, one third of SpCC originated from respiratory system. The age and site distribution are shown in Figure 1. The basic demographic and clinico-pathologic characteristics of the whole cases are summarized in Table 1.

**Table 1:** The basic demographic and clinico-pathologic characteristics of SpCC patients

Demographic and clinico-pathologic parameters		Disease specific survival				Overall survival			
		Alive	Dead	Total	*P*-value	Alive	Dead	Total	*P*-value
**Gender**	Female	216	666	882	**0.005**	271	874	1145	**0.001**
	Male	173	738	911		213	978	1191	
**Age**	≤ 69 years	271	661	932	**0.000**	318	794	112	**0.000**
	> 69 years	118	743	861		166	1058	1224	
**Race**	White	307	1156	1463	**0.027**	390	1552	1542	0.095
	Black	41	153	194		48	190	238	
	Others	42	94	136		46	110	156	
**Tumorlocation**	Digestive system	152	361	513	**0.000**	110	231	331	**0.000**
	Respiratory system	105	729	834		94	590	684	
	Endocrine system	15	131	146		10	105	115	
	Reproductive system	125	184	1969		100	161	261	
	Urinary system	30	131	161		28	80	108	
	Other site	16	22	38		6	11	17	
	Unknown	41	294	335		41	226	267	
**T stage**	TX+ T0	42	244	286	**0.000**	54	312	366	**0.000**
	T1	104	72	176		135	105	240	
	T2	81	192	273		100	265	365	
	T3	41	135	176		50	163	213	
	T4	26	213	239		34	288	322	
**N stage**	NX+N0	262	596	858	**0.000**	335	802	1137	**0.000**
	N1	17	80	97		20	109	129	
	N2	13	152	165		15	188	203	
	N3	2	29	31		3	35	38	
**M stage**	MX	13	48	61	**0.000**	18	75	93	**0.000**
	M0	264	462	726		335	620	955	
	M1	17	347	364		20	437	457	
**AJCC stage**	I stage	119	96	215	**0.000**	137	86	223	**0.000**
	II stage	73	103	176		96	130	226	
	III stage	37	157	194		30	108	138	
	IV stage	25	263	288		33	349	382	
**SEER historic stage**	Localized	233	265	498	**0.000**	289	380	669	**0.000**
	Regional	73	229	302		83	418	501	
	Distant metastasized	24	494	518		31	636	667	
**Pathologic grade**	Grade I	22	11	33	**0.000**	27	18	45	**0.000**
	Grade II	23	27	50		34	34	68	
	Grade III	82	367	449		99	479	578	
	Grade IV	40	224	264		51	278	329	
**Treatment**	Surgery alone	209	343	552	**0.000**	262	488	750	**0.000**
	Radiotherapy	34	302	336		44	375	419	
	Surgery with radiotherapy	83	215	298		98	263	361	

**Figure 2:**
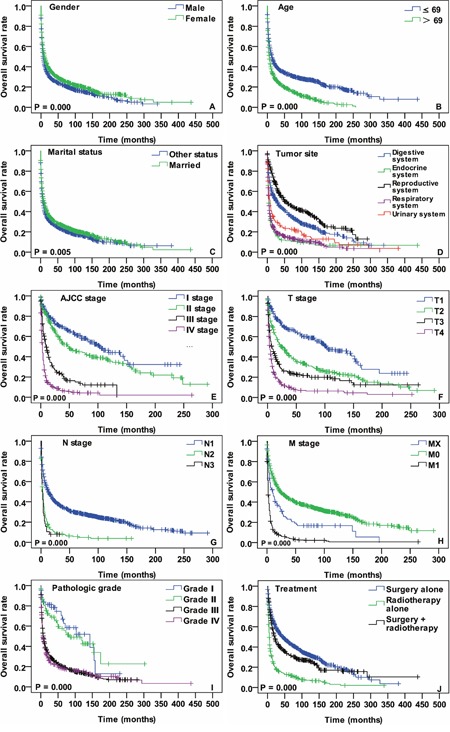
OS curves of cases with SpCC compared according to **(A)** gender, **(B)** age, **(C)** marital status, **(D)** tumor location, **(E)** AJCC stage, **(F)** T stage, **(G)** N stage, **(H)** M stage, **(I)** pathologic grade and **(J)** treatment modalities. Log-rank test was utilized to compare curves, and significance is presented on each panel.

Kaplan-Meier analysis was performed for time-to-event analysis for OS. Significant OS differences were identified depending on gender (*P* = 0.000), age (*P* = 0.000), marital status (*P* = 0.005), tumor site (*P* = 0.000), AJCC stage (*P* = 0.000), T stage (*P* = 0.000), N stage (*P* = 0.000), M stage (*P* = 0.000), pathologic grade (*P* = 0.000) and treatment modality (*P* = 0.000) (Figure 2). Univariate and multivariate survival analysis using the cox proportional hazards regression model were performed. In the univariate analysis, gender, age, marital status, primary tumor site, AJCC stage, T stage, N stage, M stage, pathologic grade and treatment modality were associated with OS. Details of these analysis are shown in Table 2. More importantly, the age > 69 years [HR 95% CI: 1.427 (1.090-1.868), *P* = 0.01, age ≤ 69 years - as Ref], tumor location in respiratory system [HR 95% CI: 1.550 (1.001-2.400), *P* = 0.04, digestive system - as Ref], N2 stage [HR 95% CI: 1.962 (1.209-3.181), *P* = 0.006, stage N0 - as Ref], AJCC stage III [HR 95% CI: 2.242 (1.106-4.545), *P* = 0.025, stage I - as Ref] and AJCC stage IV [HR 95% CI: 4.601 (2.084-10.160), *P* = 0.000, stage I - as Ref] were independently associated with worse OS (Table 3).

**Table 2:** Univariate Cox regression analysis of characteristics associated with disease specific survival and overall survival

Parameters		Disease specific survival		Overall survival	
		HR (95% CI)	*P*-value	HR (95% CI)	*P*-value
**Gender**	Male	1.00 Reference	**0.003**	1.00 Reference	**0.000**
	Female	0.854 (0.768-0.948)		0.841 (0.767-0.921)	
**Age**	≤ 69 years	1.00 Reference	**0.000**	1.00 Reference	
	> 69 years	1.618 (1.455-1.800)		1.605 (1.462-1.763)	**0.000**
**Marital status**	Other status	1.00 Reference		1.00 Reference	
	Married	0.877 (0.790-0.975)	**0.015**	0.880 (0.803-0.965)	**0.006**
**Tumor location**	Digestive system	1.00 Reference		1.00 Reference	
	Endocrine system	2.365 (1.874-2.984)	**0.000**	2.150 (1.758-2.630)	**0.000**
	Reproductive system	0.791 (0.646-0.967)	**0.022**	0.716 (0.599-0.855)	**0.000**
	Respiratory system	1.951 (1.673-2.276)	**0.000**	2.024 (1.781-2.300)	**0.000**
	Urinary system	1.384 (1.071-1.789)	**0.013**	1.525 (1.247-1.864)	**0.000**
**AJCC stage**	Stage I	1.00 Reference		1.00 Reference	
	Stage II	1.418 (1.075-1.871)	**0.014**	1.414 (1.087-1.841)	**0.010**
	Stage III	3.266 (2.526-4.223)	**0.000**	3.511 (2.662-4.632)	**0.000**
	Stage IV	6.904 (5.406-8.818)	**0.000**	7.026(5.556-8.884)	**0.000**
**T stage**	T1	1.00 Reference		1.00 Reference	
	T2	2.014 (1.536-2.642)	**0.000**	1.951 (1.555-2.446)	**0.000**
	T3	3.036 (2.296-4.085)	**0.000**	2.950 (2.305-3.776)	**0.000**
	T4	5.337 (4.062-7.014)	**0.000**	5.014 (3.990-6.302)	**0.000**
**N stage**	N0	1.00 Reference		1.00 Reference	
	N1	1.472 (1.163-1.863)	**0.001**	1.485 (1.215-1.816)	**0.000**
	N2	2.525(2.104-3.030)	**0.000**	2.424 (2.061-2.850)	**0.000**
	N3	2.972 (2.039-4.331)	**0.000**	2.892 (2.055-4.070)	**0.000**
**M stage**	MX	1.00 Reference		1.00 Reference	
	M0	0.649 (0.482-0.875)	**0.004**	0.591 (0.465-0.752)	**0.000**
	M1	2.730 (2.011-3.706)	**0.000**	2.356 (1.840-3.018)	**0.000**
**Pathologic grade**	G1	1.00 Reference		1.00 Reference	
	G2	1.620 (0.804-3.268)	0.177	1.220 (0.689-2.161)	0.0495
	G3	3.823 (2.097-6.968)	**0.000**	3.164 (1.975-5.068	**0.000**
	G4	4.092 (2.232-7.502)	**0.000**	3.367 (2.089-5.428)	**0.000**
**Treatment**	Surgery alone	1.00 Reference		1.00 Reference	
	Radiotherapy alone	2.814 (2.402-3.296)	**0.000**	2.725 (2.374-3.128)	**0.000**
	Surgery + radiotherapy	1.300 (1.095-1.542	**0.000**	1.236 (1.064-1.436)	**0.006**

**Table 3:** Multivariate Cox regression analysis of characteristics associated with disease specific survival and overall survival

Parameters		Disease specific survival		Overall survival	
		HR (95% CI)	*P*-value	HR (95% CI)	*P*-value
**Age**	≤ 69 years	1.00 Reference		1.00 Reference	
	> 69 years	1.491 (1.149-1.935)	**0.003**	1.427 (1.090-1.868)	**0.010**
**Tumor location**	Digestive system	1.00 Reference		1.00 Reference	
	Respiratory system	1.561 (1.009-2.415)	**0.040**	1.550 (1.001-2.400)	**0.041**
**AJCC stage**	Stage I	1.00 Reference		1.00 Reference	
	Stage III	-		2.242 (1.106-4.545)	**0.025**
	Stage IV	5.107 (2.563-10.173)	**0.000**	4.601 (2.084-10.160)	**0.000**
**N stage**	N0	1.00 Reference		1.00 Reference	
	N2	1.982 (1.241-3.165)	**0.004**	1.962 (1.209-3.181)	**0.006**

There were 1793 cases available for diseases specific survival (DSS) analysis in the total cases. The median follow-up time was 34.2 months (range, 1-381 months) for these cases. Nearly two fifths of cases occurred in the respiratory system (38.2%, 685/1793). The median age was 69 years and cases with age ≤ 69 years account for 52%. The early AJCC stage cases (stage I + II) were 391 and advanced stage (stage III + IV) cases 482. Most of the cases were pathologically poorly differentiated or undifferentiated carcinoma. Nearly one third of the cases were treated by surgery alone. The detailed demographic and clinico-pathologic characteristics of SpCC cases with DSS status are presented in the Table 1.

**Figure 3:**
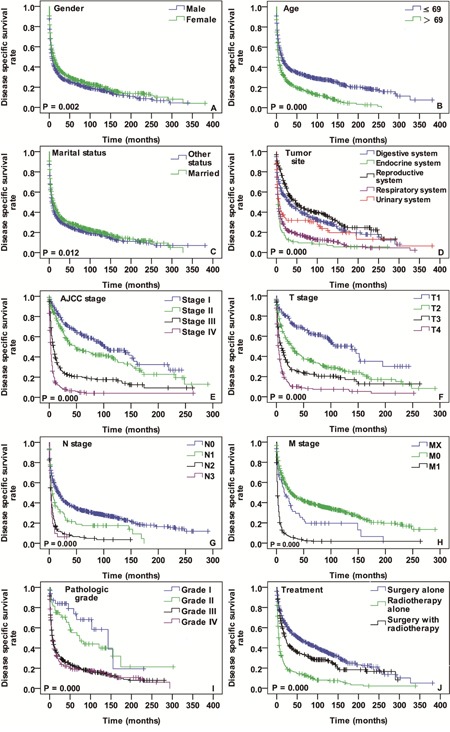
DSS curves of cases with SpCC compared according to **(A)** gender, **(B)** age, **(C)** marital status, **(D)** tumor location, **(E)** AJCC stage, **(F)** T stage, **(G)** N stage, **(H)** M stage, **(I)** pathologic grade and **(J)** treatment modalities. Log-rank test was utilized to compare curves, and significance is presented on each panel.

The DSS analysis was performed as OS was done. Significant DSS differences were found depending on gender (*P* = 0.002), age (*P* = 0.000), marital status (*P* = 0.0126), primary tumor site (*P* = 0.000), AJCC stage (*P* = 0.000), T stage (*P* = 0.000), N stage (*P* = 0.000), M stage (*P* = 0.000), pathologic grade (*P* = 0.000) and treatment (*P* = 0.000) (Figure 3). In the univariate Cox regression model, gender, age, marital status, primary tumor site, AJCC stage, T stage, N stage, M stage, pathologic grade and treatment modality were correlated with DSS (Table 2). In the multivariate analysis, age [*P* = 0.003, HR 95% CI: 1.491 (1.149-1.935) for > 69 years; ≤ 69 years - as Ref], tumor location [*P* = 0.04, HR 95% CI: 1.561 (1.009-2.415) for respiratory system; digestive system - as Ref], N stage [*P* = 0.004, HR 95% CI: 1.982 (1.241-3.165) for N2 stage; N0 stage - as Ref] and AJCC stage [*P* = 0.000, HR 95% CI: 5.107 (2.563-10.173) for stage IV; stage I - as Ref] were independent prognostic parameters for DSS. Details of the multivariate Cox proportional hazards regression analysis are shown in Table 3.

## DISCUSSION

Except for sporadic case reports and small retrospective case series, there is no adequate data to describe SpCC demographics [13]. Current study results demonstrate a peak incidence of SpCC in the seventh decades of life and the male-to-female ratio is almost 1:1 without predominance in any gender. The typical incidence of SpCC was from fifth to seventh decades in majority of the previous reports [3–5]. The most studies about SpCC are based on single-center experience and their study population is less than fifty cases. Because of the small sample size, those studies are often not sufficiently powerful to find considerable differences in survival analysis regarding to general demographic parameter such as age, gender, etc. In this survival analysis, remarkable differences were identified in both age and gender regarding DSS and OS (Figures 2A, 2B, 3A and 3B). One of the most important findings in SpCC demographics is that age is an independent prognostic indicator for DSS and OS.

More than one thousand reports are available about SpCC with variety of tumor location in the PubMed to date. Well consisted with previous reports, SpCC almost occur at any site of the body in present investigation. For better characterization, the tumor locations were categorized into five groups according to the distribution of primary tumor site: (a) respiratory system, (b) digestive system, (c) endocrine system, (d) reproductive system and (e) urinary system. In the survival comparison of these five groups, significant survival differences were found in both DSS and OS (Figures 2D and 3D). The endocrine system, respiratory system and urinary system were unfavorably associated with DSS and OS of SpCC patients (Digestive system - as a ref) in univariate Cox model. Furthermore, the respiratory system (Digestive system - as a ref) was another adverse independent prognostic indicator not only for DSS but also for OS.

Much like other carcinomas, the AJCC staging of SpCC is a valuable tool for physicians in treatment planning and prognosis evaluation [14]. The AJCC staging data of SpCC was incomplete and two thirds of them were missing in the SEER database. Survival analysis was performed with available data. There were typical survival differences in T stage, N stage, M stage and AJCC stage (Figures 2 and 3). Among them, only N stage and AJCC stage were independent prognostic parameters for DSS and OS of SpCC (Table 3).

It has been widely investigated and recognized that SpCC has worse prognosis than SCC [15]. Intrinsic tumor properties and lack of standard treatment guidelines may be the possible explanations for the unfavorable prognosis. However, SpCC showed site specific prognosis [16–18]. In this study we compared obtainable treatment modalities (Figures 2J and 3J). The results demonstrate that the surgery alone group had better DSS and OS than radiotherapy alone and surgery combined with radiotherapy group. Although this is not a standardized treatment comparison, it likely indicates that radiotherapy alone is the least option for SpCC treatment. Besides, it is difficult to draw firm conclusion that SpCC patients cannot benefit from radiotherapy according to the results in this study because there were many other confounding factors, such as unclear surgical margin, advanced tumor stage, pathologic grade, etc. Therefore, it is urgently necessary to reassess the effectiveness of radiotherapy for SpCC with multi-center well-controlled studies.

A few limitations of SEER registry and current investigation itself should be acknowledged. Firstly, not all cases have complete information, data on important parameters such as treatment modalities, pathologic grade and TNM/AJCC stage are incomplete. Secondly, due to the lack of chemotherapy data, the role of chemotherapy for SpCC could not be established. Thirdly, the median follow-up time was 32.5 months for OS and 34.2 months for DSS. The relatively short follow up time may not reveal longterm survival differences. Lastly, the retrospective nature of current investigation may have introduced bias into the overall analysis.

In conclusion, the main interest of this study is to give audience concise and generalized information about SpCC. Thus, we did not demonstrate many possible subgroup analysis and develop detailed discussions. Although SEER database are capable of providing the largest data about SpCC, those available date is still incomplete. Despite the limitation of the incomplete data and study itself, the present investigation is the first of its kind with the largest study population from multiple centers to define the overall demographic trends, basic clinico-pathologic characteristic, management and prognosis of SpCC. The investigation results demonstrate that SpCC mostly occurred at 70~80 years old in white people and sex distribution was almost equal. The patient's age, primary tumor location, N stage and AJCC stage were independent prognostic indicators for both DSS and OS.

## MATERIALS AND METHODS

International Classification of Diseases for Oncology codes (ICD-O-3) for spindle cell carcinoma (8032/3) was used for identification of cases with a diagnosis of SpCC registered in the SEER database and SEER*Stat software, version 8.3.2, was applied for data extraction.

For statistical analysis, the following demographic and clinico-pathologic parameters were selected: age, gender, race, marital status, primary tumor site, American joint committee on cancer (AJCC) stage, pathologic grade, treatment modality, follow-up time and outcome status. Not all of the cases that we identified contained all these data. Statistical analysis was performed by using the software of the Statistical Package for Social Sciences, version 23.0, for Windows (SPSS, Chicago, IL). Differences in the numerical parameters were evaluated by using the Student's test or non-parametric Wilcoxon test and the chi square test or Fisher exact test was used for categorical variables comparison. The survival curves were generated by using the Kaplan-Meier method, and the log-rank test was performed to evaluate the survival difference. Adjusted hazard ratios (HRs) along with 95% confidence intervals (CIs) were calculated by using the Cox proportional hazards regression model. When the P-value was < 0.05, the difference was regarded as statistically significant. All statistical tests were two tailed.
